# Effects of Inhalation of Essential Oil of *Citrus aurantium* L. var. *amara* on Menopausal Symptoms, Stress, and Estrogen in Postmenopausal Women: A Randomized Controlled Trial

**DOI:** 10.1155/2014/796518

**Published:** 2014-06-12

**Authors:** Seo Yeon Choi, Purum Kang, Hui Su Lee, Geun Hee Seol

**Affiliations:** Department of Basic Nursing Science, School of Nursing, Korea University, 145 Anam-ro, Seongbuk-gu, Seoul 136-701, Republic of Korea

## Abstract

This study aimed to investigate the effects of inhalation of the essential oil of *Citrus aurantium* L. var. *amara* (neroli oil) on menopausal symptoms, stress, and estrogen in postmenopausal women. Sixty-three healthy postmenopausal women were randomized to inhale 0.1% or 0.5% neroli oil or almond oil (control) for 5 minutes twice daily for 5 days. Menopause-related symptoms, as determined by the Menopause-Specific Quality of Life Questionnaire (MENQOL); sexual desire visual analog scale (VAS); serum cortisol and estrogen concentrations, blood pressure, pulse, and stress VAS, were measured before and after inhalation. Compared with the control group, the two neroli oil groups showed significant improvements in the physical domain score of the MENQOL and in sexual desire. Systolic blood pressure was significantly lower in the group inhaling 0.5% neroli oil than in the control group. Compared with the control group, the two neroli oil groups showed significantly lower diastolic blood pressure and tended to improve pulse rate and serum cortisol and estrogen concentrations. These findings indicate that inhalation of neroli oil helps relieve menopausal symptoms, increase sexual desire, and reduce blood pressure in postmenopausal women. Neroli oil may have potential as an effective intervention to reduce stress and improve the endocrine system.

## 1. Introduction

The World Health Organization (WHO) defines menopause as permanent cessation of menstruation caused by the loss of ovarian follicular activity. Menopause is a natural process of female aging and accompanies diverse physiological changes in women. One of the most prominent changes is a decrease in female sex hormones, resulting in vasomotor symptoms such as hot flushes and palpitations [1]. In addition, psychological changes, including depression, anxiety, restlessness, and sleep disorders may occur during the menopausal transition. Climacteric women may experience other symptoms, including decreased sexual desire and increased muscle pains, and carry higher risks of cardiovascular diseases. Menopausal symptoms have also been reported to have a negative impact on women's daily lives and even degrade their quality of life [2].

As vasomotor symptoms including hot flushes are primarily caused by a decrease in estrogen [3], hormone replacement therapy (HRT) has been regarded as an effective remedy [1]. Despite its positive effects, HRT has various potential side effects, including thromboembolism, gallstones, breast cancer, and stroke [4]. Moreover, HRT was found to improve quality of life only when applied for a short time [5]. Lifestyle modifications, regular exercise, and use of antidepressants have therefore been recommended in tandem with HRT [6, 7], with several recent studies analyzing the ability of complementary and alternative medicines to relieve menopausal symptoms [8].

The essential oil of* Citrus aurantium* L. var.* amara*, also known as neroli oil, has been reported to have antianxiety effects by regulating 5-HT receptors in rats [9] and to have antidepressant effects through the monoaminergic system in mice [10]. Neroli oil has also been reported to have sedative, antianxiety, and antidepressant effects on mice [11]. In addition, limonene, one of the major chemical components in the essential oil of* Citrus aurantium* L. var.* amara*, has been shown to have antianxiety [12] and motor relaxant effects, indicating sedative activity [13] in mice. Moreover, a study in rats reported that olfactory stimulation with grapefruit oil, which is rich in limonene, stimulated sympathetic nerves by activating histamine H1 receptors and that limonene treatment induced similar responses [14]. Limonene-rich bergamot essential oil also demonstrated direct vasorelaxant effects [15].

Taken together, these findings in rodents indicate that neroli oil can be effective in relieving not only psychological climacteric symptoms, such as stress, depression, and anxiety, but also physiological symptoms, such as those related to vasomotor effects and blood pressure. To date, however, no clinical study has tested the effects of neroli oil on postmenopausal women. This study therefore evaluated the effects of neroli oil inhalation on physiological and psychological symptoms in postmenopausal women and assessed the potential of neroli oil inhalation as a nursing intervention.

## 2. Materials and Methods

### 2.1. Study Design and Participants

This double-blinded, randomized controlled trial was designed to assess the effects of inhalation of several concentrations of neroli oil on menopausal symptoms, stress, and estrogen levels in healthy postmenopausal women. Ninety-two women aged ≤65 years with natural menopause living in Seoul, South Korea, were recruited for the study between November 2013 and March 2014; of these women, 11 did not meet the eligibility criteria or withdrew their consent to participate. The remaining 81 participants were told the purpose and protocol of the experiment. The detailed inclusion criteria included (1) age ≤65 years with natural menopause, (2) no experience of HRT or aromatherapy in the previous six weeks, (3) no history of psychiatric illness, (4) no current medication for anxiety or depression, (5) no disturbance of olfactory acuity, and (6) being free of allergies related to aromatherapy. Eighteen participants were excluded, including 11 who missed two or more treatments with neroli or almond oil, five who were not assessed after treatment, and two who used drugs such as antibiotics during the treatment period. The study design and protocol were approved by the Ethical Review Committee of the Chung-Ang University (code: 1041078-201310-HR-0072-03), and all participants provided written informed consent.

### 2.2. Intervention

Neroli oil and almond oil were obtained from Aromarant Co. Ltd. (Rottingen, Germany). Neroli oil was dissolved in almond oil that has no smell and no deleterious effects, at concentrations of 0.1% and 0.5% (v/v). The participants were assigned by a random number table to groups receiving 0.1% or 0.5% (v/v) neroli oil dissolved in almond oil, or almond oil (control). Only the compounder was aware of subject assignment. Neither the participants nor the investigators knew the allocation. Subjects in the three groups received 10 bottles, each containing 1 mL of neroli oil in almond oil or almond oil; all bottles had the same shape and color. Each subject was self-treated for 10 sessions, performed at 10 AM and 10 PM for 5 consecutive days. Each subject was instructed to decant the contents of one bottle onto a fragrance pad, sit in a stable and comfortable place, position the pad 30 cm away from her nose, and inhale the fragrance for five minutes with normal breathing.

### 2.3. Outcome Measurements and Data Collection

Pretrial surveys and measurements were performed on the day before day 1 of the 5-day intervention, while posttrial surveys and measurements were performed on day 6. The pretrial survey included general characteristics, self-reported menopause-specific qualify of life questionnaire (MENQOL), and stress and sexual visual analog scale (VAS), while pretrial measurements included systolic blood pressure (SBP), diastolic blood pressure (DBP), pulse rate, and serum cortisol and estrogen levels. Posttrial survey and measurements included all of the above parameters, except for general characteristics. To minimize the effect of data collection time, all surveys and measurements were conducted between 9 AM and 10 AM.

#### 2.3.1. MENQOL

The MENQOL is a self-reported survey, composed of 29 assessment items, including 16 in the physical domain, 7 in the psychosocial domain, 3 in the vasomotor domain, and 3 in the sexual functioning domain. For each item, the respondent selects whether or not she experienced that symptom over the previous four weeks; if yes, she rates it on a scale from 2, corresponding to “not bothered at all,” to 8, corresponding to “extremely bothered” [16]. Mean total MENQOL score and mean score on each domain were determined. A factor analysis verified the reliability of the MENQOL and showed that five questions were redundant [17]. This study therefore excluded the five redundant questions and assessed the remaining 24 questions. The present study used a Korean translation of the original MENQOL, which has been used in previous research after an expert review [18].

#### 2.3.2. Sexual Desire Scale and Stress Scale

The sexual desire of each participant was measured by VAS [19], a 10 cm line oriented horizontally. Each participant marked on the scale by crossing the line at the point that corresponded to the intensity of sexual desire, with 0 indicating no sexual desire and 10 indicating extremely strong desire. The same VAS was used for subjective measurement of stress level. Each participant marked the intensity of stress, where 0 corresponded to no stress and 10 to extreme stress.

#### 2.3.3. Blood Pressure and Pulse Rate

The SBP and DBP of each participant were measured with a sphygmomanometer on the left arm after 15 minutes of rest in a sitting position. Both before and after the intervention, SBP and DBP were measured twice and their averages were calculated. Pulse rate per minute was measured twice with a stop watch before and after the intervention after 15 minutes of rest in a sitting position, and the averages were calculated.

#### 2.3.4. Serum Cortisol and Estrogen Concentrations

Blood samples (3 mL each) were obtained before and after the intervention from each subject between 9 and 10 AM. Serum was obtained by centrifugation and was immediately frozen and stored at −70°C. Serum cortisol (Enzo Life Sciences, NY, USA) and estrogen (CUSABIO, Wuhan, China) concentrations were measured by ELISA, with the results read in a microplate reader at a wavelength of 450 nm.

### 2.4. Statistical Analysis

All data are reported as mean ± standard deviation, with all statistical analyses performed using SPSS version 20.0 (SPSS Inc., Chicago, IL, USA). Intergroup comparisons of any normally distributed variable were performed using one-way analysis of variance (ANOVA), followed by least significant difference Scheffe's posthoc analysis. Nonnormally distributed variables were compared using the Kruskal-Wallis test, with posthoc analysis by the Mann-Whitney test and Bonferroni's method for significance threshold adjustment. Within group, comparisons of normally and nonnormally distributed variables were assessed using paired *t*-tests and Wilcoxon signed rank tests, respectively. A *P* value <0.05 was defined as statistically significant.

## 3. Results

### 3.1. General Characteristics of the Participants and Test of Homogeneity

**Figure 1:**
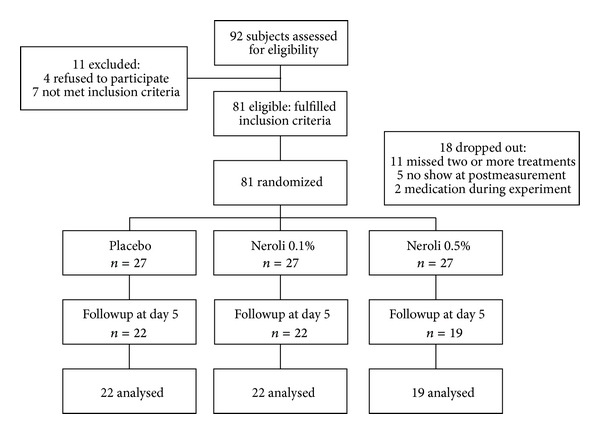
Study flow diagram.

Of the 92 women assessed for eligibility, seven did not meet the eligibility criteria and four refused to participate. Of the remaining 81 women, 18 dropped out during the study period, including 11 who missed two or more treatment sessions, 5 who did not attend posttreatment measurements, and 2 who were on medication during the intervention. Thus, data were collected and analyzed for 63 women, including 22 who received almond oil, 22 who received 0.1% neroli oil, and 19 who received 0.5% neroli oil (Figure 1). The mean age of the study population was 55.81 years and their mean BMI was 22.97 kg/m^2^. There were no significant differences among the three groups in general characteristics, including age and BMI, or in any of the baseline outcome measures (Table 1).

### 3.2. Effect of Neroli Oil on Menopausal Symptoms, Sexual Desire, and Stress

After the 5 days of intervention, the total MENQOL score significantly decreased from the baseline in the 0.1% (*P* < 0.001) and 0.5% (*P* = 0.03) neroli oil groups but did not decrease significantly in the control group (Table 2).

The three groups showed significant differences in mean change of the physical domain of the MENQOL (*P* = 0.04, Table 2). A posthoc analysis showed a significant difference between the 0.1% neroli oil and control groups (*P* = 0.008). The mean changes in the other domains did not differ significantly among the groups but tended to be more pronounced in the two neroli oil groups than in the almond oil group. The 0.1% neroli oil group showed a significant change in mean physical domain (*P* < 0.001) and vasomotor domain (*P* = 0.002) scores.

Although the sexual desire VAS score of the control group decreased significantly (*P* = 0.013) after the intervention, the sexual desire VAS scores of the 0.1% (*P* = 0.049) and 0.5% (*P* = 0.001) neroli oil groups increased significantly, with the three groups differing significantly in mean change (*P* < 0.001). A posthoc analysis based on the Mann-Whitney test showed that both the 0.1% (*P* = 0.001) and 0.5% (*P* < 0.001) neroli oil groups had significantly higher sexual desire VAS scores after treatment than the control group.

Stress level, as assessed by stress VAS measurement, decreased in all three groups but did not differ significantly among the groups.

### 3.3. Effect of Neroli Oil on Blood Pressure and Pulse Rate

**Figure 2:**
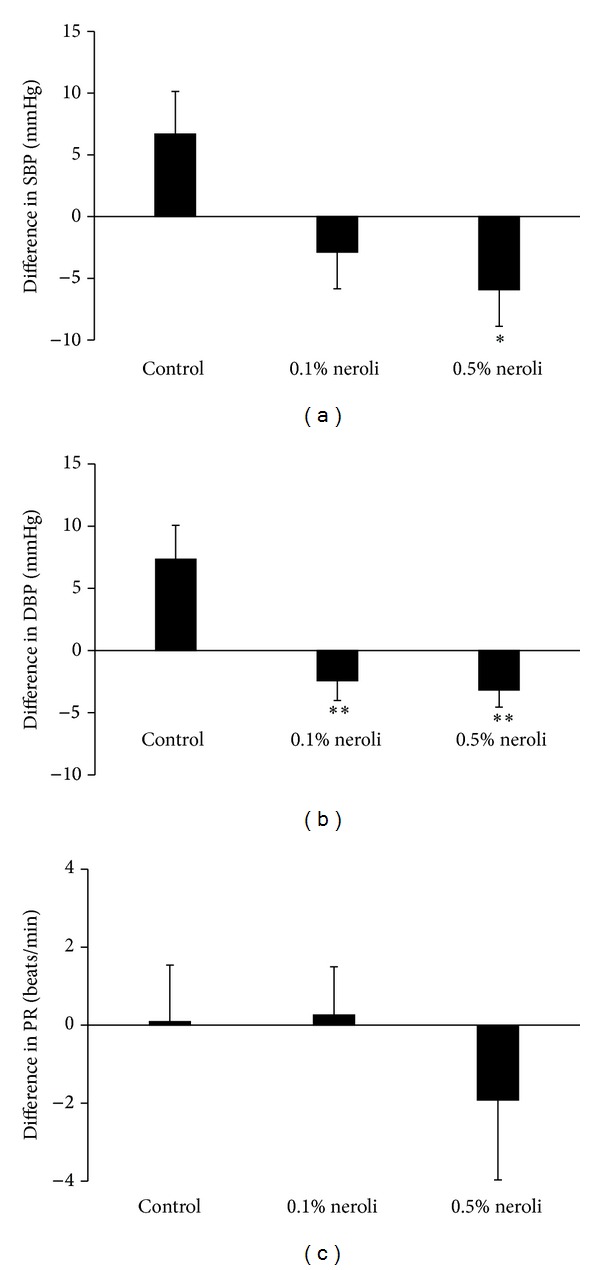
Effects of neroli oil inhalation on (a) systolic blood pressure, (b) diastolic blood pressure, and (c) pulse rate. Values are expressed as mean ± SEM. *P < 0.05, **P < 0.01 compared with the control group. SBP, systolic blood pressure; DBP, diastolic blood pressure; PR, pulse rate.

After the 5-day intervention, the SBP of the control group increased 6.68 ± 16.23 mmHg, whereas the SBPs of the 0.1% and 0.5% neroli oil groups decreased 2.89 ± 13.89 mmHg and 5.92 ± 12.90 mmHg, respectively, with the difference in mean changes among the groups being statistically significant (*P* = 0.03, Figure 2(a)). A posthoc analysis showed a significant difference between the 0.5% neroli oil and control groups (*P* = 0.03).

In addition, DBP of the control group increased 7.34 ± 12.78 mmHg, whereas the DPBs of the 0.1% and 0.5% neroli groups decreased 2.43 ± 7.47 mmHg and 3.18 ± 5.97 mmHg, respectively, with the difference in mean changes among the groups being statistically significant (*P* = 0.001, Figure 2(b)).

Pulse rate increased in the control (0.09 ± 6.80 beats/min) and 0.1% neroli oil (0.26 ± 5.80 beats/min) groups but decreased −1.92 ± 8.93 beats/min in the 0.5% neroli oil group, with none of these differences being statistically significant (Figure 2(c)).

### 3.4. Effect of Neroli Oil on Serum Cortisol and Estrogen Levels

After the 5-day treatment period, serum cortisol concentrations decreased in all three groups, but none of the differences was statistically significant (Table 3). Similarly, there were no differences in serum estrogen concentrations within or among the three groups (Table 4).

## 4. Discussion

This study was designed to investigate the effects of neroli oil inhalation on menopausal symptoms, stress, and serum estrogen level among postmenopausal women. Postmenopausal women aged ≤65 years inhaled neroli oil or almond oil twice daily for five days, and the effects on MENQOL, sexual desire and stress VAS, blood pressure, pulse rate, and serum cortisol and estrogen concentrations were measured.

The MENQOL measures the quality of life related to climacteric symptoms among women in menopausal transition. This study showed that inhalation of neroli oil, at concentrations of 0.1% and 0.5%, significantly altered total MENQOL score and significantly enhanced sexual desire, as measured by a VAS, compared with the inhalation of almond oil alone. In contrast, the sexual domain of the MENQOL was not improved significantly in the neroli oil groups. A previous study of the MENQOL reported that the four domains—vasomotor, physical, psychosocial, and sexual functioning—have correlations among themselves [20]. In addition, the sexual functioning domain of the MENQOL assessed functional aspects such as vaginal dryness, as well as subjective aspects. The sexual desire VAS measured only subjective aspects. Therefore, the significant improvements in the physical and vasomotor domains in the two neroli oil groups presumably affected sexual desire VAS, although the sexual domain of the MENQOL itself did not change significantly.

Women in menopausal transition frequently experience both vasomotor and psychological symptoms, but both have different underlying factors. Psychological symptoms are associated with subject lifestyle and behavioral factors, whereas vasomotor symptoms show much stronger correlations with menopausal stages than with lifestyle and environmental elements [21]. In addition, decreased estrogen is an important factor in the complex mechanism of hot flushes [22]. The present study showed that vasomotor symptoms improved significantly after treatment with 0.1% neroli oil. Neurotransmitters such as 5-HT are involved in regulating body temperature; using the same mechanism, HRT activates noradrenaline or neurotransmitters like 5-HT to treat vasomotor symptoms [22]. The significant improvements in vasomotor symptoms observed in subjects who inhaled neroli oil were likely due to the activation of 5-HT neurotransmitters, a mechanism similar to the antianxiety effects of neroli oil in animal models [9].

Stress poses significant risks in cardiovascular diseases, and an increased level of stress can cause physiological responses by the cardiovascular system, including increases in blood pressure and pulse rate [23]. Although the present study did not show a significant change in subjective stress VAS among the three groups, neroli oil inhalation significantly reduced both SBP and DBP, suggesting that neroli oil relieves cardiovascular responses to stress. In addition, although the concentration of serum cortisol, a physiological indicator of stress, did not decrease in either of the neroli oil groups, the decrease was greater in the 0.5% neroli oil group than in the 0.1% neroli oil and control groups. This result is in agreement with findings showing that neroli oil had sedative and relaxant effects in animals [13] and that essential oil from bergamot, another member of the citrus family, not only lowered blood pressure but also relaxed blood vessels in animals [15, 24]. Although serum cortisol levels did not differ among the three groups, the results presented here suggest that neroli oil reduces physiological responses to stress.

Neroli oil may also reduce blood pressure by acting on the autonomic nervous system. Olfactory stimulation with limonene, which is abundant in neroli essential oil, was found to elevate sympathetic nerve activity by activating H1 receptors in an animal model [14]. Moreover, neroli oil has been reported to contain adrenergic amines such as synephrine, octopamine, and tyramine [25]. These results suggest that neroli oil treatment modulates autonomic nerves to reduce blood pressure.

Herbal remedies for treatment of climacteric symptoms include black cohosh, hops, wild yam, and ginseng [8]. Terpenes, composed of isoprene as a building block, can be found in most of these herbs, as well as in neroli oil. Although serum estrogen levels did not increase significantly after neroli oil treatment, serum estrogen slightly increased in the 0.5% neroli oil group while slightly decreasing in the other two groups. Similar to other herbs, neroli oil may affect the endocrine system in alleviating menopausal symptoms.

## 5. Conclusion

In summary, the present randomized controlled trial showed that inhalation of neroli oil by postmenopausal women improved their quality of life related to menopausal symptoms, increased sexual desire, and reduced blood pressure. In addition, inhalation of neroli oil may reduce stress levels and stimulate the endocrine system. These findings indicate that neroli oil can be used to relieve various symptoms related to menopause.

## Figures and Tables

**Table 1:** Homogeneity test for general characteristics and measurement variables.

Characteristics or variables	Control (*n* = 22)	0.1% neroli oil (*n* = 22)	0.5% neroli oil (*n* = 19)	Total (*N* = 63)	*P* value
Age (years)	55.73 (2.35)	56.46 (2.26)	55.16 (2.99)	55.81 (2.55)	0.27
BMI (kg/m^2^)	22.75 (2.48)	22.74 (1.78)	23.49 (1.91)	22.97 (2.08)	0.43
Age at menarche (years)	15.90 (1.38)	15.59 (1.79)	15.19 (1.42)	15.57 (1.55)	0.31
Age at menopause (years)	50.05 (10.24)	52.27 (2.45)	51.53 (2.09)	51.27 (6.30)	0.50
Frequency of lifetime pregnancy	2.82 (0.85)	3.00 (1.31)	2.95 (0.85)	2.92 (1.02)	0.85^a^
Number of children	1.86 (0.47)	2.05 (0.79)	1.95 (0.52)	1.95 (0.61)	0.46^a^
Number of family members	3.32 (1.04)	3.32 (1.13)	3.37 (1.30)	3.33 (1.14)	0.94^a^
Duration of physical exercise (min/week)	188.18 (223.39)	213.75 (191.84)	255.26 (329.99)	217.34 (248.45)	0.63^a^
Frequency of physical exercise (times/week)	3.00 (3.19)	3.73 (3.01)	3.81 (2.09)	3.5 (2.81)	0.22^a^
Frequency of sexual intercourse (times/6 months)	9.87 (10.18)	8.36 (7.82)	7.87 (11.67)	8.74 (9.80)	0.54^a^
MENQOL (score)					
Overall	2.66 (0.95)	2.53 (0.91)	2.63 (1.04)	2.61 (0.95)	0.91
Physical	2.58 (1.07)	2.44 (0.29)	2.66 (1.27)	2.55 (1.06)	0.97^a^
Psychological	2.45 (1.49)	1.90 (1.01)	2.24 (1.26)	2.22 (1.27)	0.40^a^
Sexual	3.80 (1.62)	4.02 (2.27)	3.46 (1.87)	3.77 (1.93)	0.70^a^
Vasomotor	2.20 (1.69)	2.52 (1.66)	2.16 (1.59)	2.30 (1.63)	0.53^a^
Sexual desire VAS (cm)	3.61 (2.69)	2.98 (2.05)	2.74 (1.79)	3.13 (2.22)	0.62^a^
Stress VAS (cm)	4.64 (1.53)	3.86 (2.26)	4.72 (2.04)	4.39 (1.97)	0.30^a^
SBP (mmHg)	122.48 (15.65)	122.59 (11.57)	117.32 (12.89)	120.96 (13.51)	0.38
DBP (mmHg)	78.52 (9.31)	78.11 (8.35)	72.76 (8.04)	76.64 (8.85)	0.07
PR (beats/min)	71.23 (6.87)	72.04 (8.73)	72.05 (7.61)	71.76 (7.66)	0.92
Serum cortisol (ng/mL)	7.80 (4.90)	6.22 (3.85)	11.53 (15.64)	8.38 (9.43)	0.45^a^
Serum estrogen (ng/mL)	139.76 (27.92)	135.25 (21.82)	143.78 (26.64)	139.40 (25.37)	0.69^a^

BMI, body mass index; MENQOL, Menopause-Specific Qualify of Life Questionnaire; VAS, visual analog scale; SBP, systolic blood pressure; DBP, diastolic blood pressure; PR, pulse rate.

Data reported as mean (standard deviation).

One-way ANOVA, ^a^Kruskal-Wallis test.

**Table 2:** Effect of neroli oil on menopausal symptoms, sexual desire, and stress (*N* = 63).

Variables	Control (*n* = 22)	0.1% neroli oil (*n* = 22)	0.5% neroli oil (*n* = 19)	*P* value
MENQOL (score)				
Overall	−0.19 ± 0.80	−0.71 ± 0.61	−0.52 ± 0.88	0.27
Physical^a^	−0.02 ± 0.81	−0.64 ± 0.57	−0.40 ± 0.97	0.04∗
Psychological^a^	−0.28 ± 1.65	−0.28 ± 0.97	−0.43 ± 1.24	0.76
Sexual^a^	−0.74 ± 1.56	−1.50 ± 1.83	−1.39 ± 1.61	0.45
Vasomotor^a^	−0.20 ± 1.30	−0.92 ± 1.13	−0.35 ± 1.34	0.06
Sexual desire VAS^a^ (cm)	−1.82 ± 3.03	0.81 ± 1.84	3.10 ± 3.10	<0.01∗∗∗
Stress VAS^a^ (cm)	−1.52 ± 2.30	−1.08 ± 1.98	−2.28 ± 2.49	0.24

MENQOL, Menopause-Specific Qualify of Life Questionnaire; VAS, visual analog scale.

One-way ANOVA, ^a^Kruskal-Wallis test.

Data presented as mean ± standard deviation.

*P < 0.05, ***P < 0.001 compared with the control group.

**Table 3:** Effect of neroli oil on serum cortisol levels (*N* = 63).

	Before (ng/mL)	After (ng/mL)	*P* value	Difference (ng/mL)	*P* value
Control	7.80 ± 4.90	7.52 ± 8.77	0.76	−0.28 ± 6.85	0.571
0.1% neroli oil	6.22 ± 3.85	6.08 ± 2.93	0.73	−0.14 ± 4.61	
0.5% neroli oil	11.53 ± 15.64	8.41 ± 7.32	0.38	−3.12 ± 9.40	

Data presented as mean ± standard deviation.

**Table 4:** Effect of neroli oil on serum estrogen levels (*N *= 63).

	Before (ng/mL)	After (ng/mL)	*P* value	Difference (ng/mL)	*P* value
Control	139.76 ± 27.92	135.92 ± 24.00	0.49	−3.84 ± 18.60	0.270
0.1% neroli oil	135.25 ± 21.82	131.70 ± 24.37	0.43	−3.55 ± 24.11	
0.5% neroli oil	143.78 ± 26.64	148.33 ± 34.67	0.94	4.55 ± 25.78	

Data presented as mean ± standard deviation.
